# Bulk CsPbCl_*x*_Br_3-*x*_ (1 ≤ *x* ≤ 3) perovskite nanocrystals/polystyrene nanocomposites with controlled Rayleigh scattering for light guide plate

**DOI:** 10.1038/s41377-023-01306-z

**Published:** 2023-11-01

**Authors:** Chongming Liu, Zhicheng Zhu, Kaibo Pan, Yuan Fu, Kai Zhang, Bai Yang

**Affiliations:** State Key Laboratory of Supramolecular Structure and Materials, College of Chemistry, Jilin University, 130012 Changchun, China

**Keywords:** Nanoparticles, Displays

## Abstract

Perovskite nanocrystals (PNCs)/polymer nanocomposites can combine the advantages of each other, but extremely few works can achieve the fabrication of PNCs/polymer nanocomposites by bulk polymerization. We originally adopt a two-type ligand strategy to fabricate bulk PNCs/polystyrene (PS) nanocomposites, including a new type of synthetic polymerizable ligand. The CsPbCl_3_ PNCs/PS nanocomposites show extremely high transparency even the doping content up to 5 wt%. The high transparency can be ascribed to the Rayleigh scattering as the PNCs distribute uniformly without obvious aggregation. Based on this behavior, we first exploit the potential of PNCs to serve as scatters inside light guided plate (LGP), whose surface illuminance and uniformity can be improved, and this new kind of LGP is compatible with the advanced liquid crystal display technology. Thanks to the facile composition adjustment of CsPbCl_*x*_Br_3-*x*_ (1 ≤ *x* ≤ 3) PNCs, the Rayleigh scattering behavior can also be adjusted so as to the performance of LGP. The best-performing 5.0-inch LGP based on CsPbCl_2.5_Br_0.5_ PNCs/PS nanocomposites shows 20.5 times higher illuminance and 1.8 times higher uniformity in display than the control. The LGP based on PNCs/PS nanocomposite exhibits an enormous potential in commercialization no matter based on itself or combined with the LGP-related technology.

## Abstract (web summary)

High transparent perovskite nanocrystals/polystyrene nanocomposites can be fabricated by a two-type ligand strategy and have enormous potential to serve as a new kind of light guide plate.

## Graphical abstract

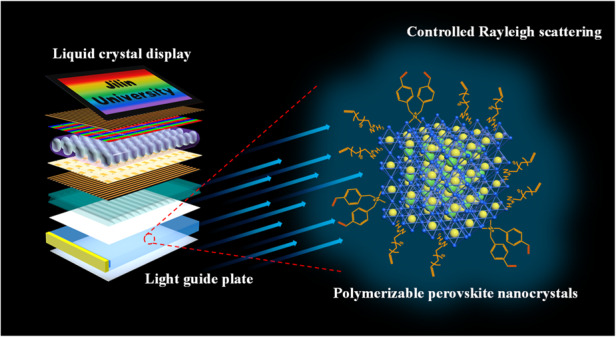

## Introduction

In recent years, perovskite nanocrystals (PNCs) have become an attractive material due to their wonderful properties such as defect tolerance, facile synthesis, high photoluminescence with narrow bandwidth and easily tunable bandgap through size or composition adjustment^1–3^. Despite the extraordinary properties of PNCs, they suffer from instability, especially when exposed to light, moisture, polar solvents, and high temperature, as their dynamic nature of the perovskite lattice and ligand binding^2,4,5^. The instability discounts their performance and limits their further application.

The fact that nanoparticle and polymer hybrid materials can often combine the advantages of each has been demonstrated in several fields^6–9^. Embedding PNCs into polymer is an effective strategy to enhance the PNCs stability and polymer can endow the PNCs with other positive effects based on different structure and functional groups, such as surface defect passivation^10^, facilitating the charge separation and transport^11^, assisted self-assembly and controlled morphology^12–14^, and excellent processability, stretchability, and mechanical property in the form of nanocomposites^15^. Therefore, PNCs/polymer nanocomposite is promising for displays^15–17^, luminescent solar concentrators^18,19^, scintillator^20,21^, lighting^22–24^ and hybrid photovoltaic device^25,26^. The uniform distribution of PNCs in polymer matrix is critical to the properties of the nanocomposites and the aggregation of PNCs induced by high surface energy has a severe influence on the performance of related applications^27–31^. As such, the loading fraction is limited owing to the phase separation between PNCs and polymer. Chemical interaction between PNCs and polymer is necessary to suppress the phase separation.

Meanwhile, most of the fabrication methods of PNCs/polymer nanocomposites are spin coating, swelling-shrinking and electrospinning based on the in situ synthesis of PNCs in polymer matrix and physical mixing^5,32^. To enhance the chemical anchoring of polymers onto PNCs, there are also quite a number of researches about the structural design of polymer with functional groups. The polymers always act as ligands or host matrices, including ammonium bromide or carboxyl-functionalized polystyren^31,33^, zwitterion-containing copolymers^10,27^, polyacrylic acid-grafted graphene oxide^34^, polyvinyl pyrrolidone^35^, carboxyl or amino-functionalized poly(ethylene glycols)^12^, poly(maleic anhydride-alt-1-octadecene)^36^, hydrolyzed poly(methyl methacrylate)/highly branched poly(ethylenimine)^37^, etc.

However, extremely few works can satisfy the bulk polymerization to fabricate nanocomposites, where polymerizable capping agents play an important role to disperse PNCs into polymer matrix uniformly. In 2017, Sun et al. first synthesized 4-vinyl-benzyl-dimethyloctadecylammonium chloride as a polymerizable ligand, whose ammonium group can interact with MAPbBr_3_ PNCs and the styryl group can help MAPbBr_3_ PNCs copolymerize with styrene and methyl methacrylate to obtain homogeneous bulk MAPbBr_3_ PNCs/polymer nanocomposites^38^. Methacrylic acid modified PNCs copolymerized with hydrophobic methyl methacrylate and methacrylisobutyl polyhedral oligomeric silsesquioxane monomers were also reported and the nanocomposites show excellent luminescent performance and stability^30,39^. Recently, Li et al. found zinc methacrylate can stabilize the CsPbBr_3_ PNCs in polymethyl methacrylate matrix by replacing the surface ammonium ligands^28^. Based on the strong ion–dipole interactions between the CF_3_ of fluorinated acrylate and the positively charged PNCs, Liu et al. fabricated luminescent PNCs/fluorinated polymers nanocomposites, exhibiting stretchable and self-healable properties and high resistance to a harsh environment^40^. Nevertheless, the loading fraction of PNCs in nanocomposites is typically at an extremely low level <1.0 wt% accompanied by slight aggregation, which means a further effective strategy is demanded for suppressing the phase separation.

Therefore, in this work, we adopted a two-type ligand strategy to fabricate PNCs/polystyrene (PS) nanocomposites, where the undec-10-en-1-amine (EAm) help PNCs disperse in styrene and the synthetic bis[(4-ethenylphenyl)methyl]dimethylammonium chloride (BEMDA) works as polymerizable capping ligands to endow the PNCs with polymerization activity. The bulk CsPbCl_3_ PNCs/PS nanocomposites can still maintain high transparency even at the doping content up to 5 wt%. We ascribed the high transparency to the uniform distribution of PNCs in PS matrix, which means the scattering of light can be described by Rayleigh theory. Additionally, the high refractive index of perovskite materials has been reported in recent years^41–44^, while the potential effects and applications related to their high refractive have not drawn much attention as their photovoltaic and luminescent properties did^42,45,46^. We focus on the Rayleigh scattering inside the nanocomposites caused by the higher refractive index of PNCs than PS. We found PNC can be a type of scatter inside PS-type light guide plate (LGP) enhancing the on-surface illuminance and uniformity. We also systematically studied the mechanism of the improved surface illuminance and the influence of different PNC compositions CsPbCl_*x*_Br_3-*x*_ (1 ≤ *x* ≤ 3) on the performance of LGP. The refractive index of PNCs can be adjusted from 1.70 to 2.02 at 450 nm for CsPbCl_*x*_Br_3-*x*_ (1 ≤ *x* ≤ 3) PNCs. For 5.0-inch LGP, the best performing LGP doping with 1 wt% CsPbCl_2.5_Br_0.5_ PNCs exhibits about 20.5 times higher illuminance and 1.8 times higher uniformity in display than the control. This new kind of LGP based on the PNCs/PS nanocomposite is compatible with advanced liquid crystal display (LCD) technology, indicating great value in practical application.

## Results

### Facile production of styrene-soluble PNCs

**Fig. 1:**
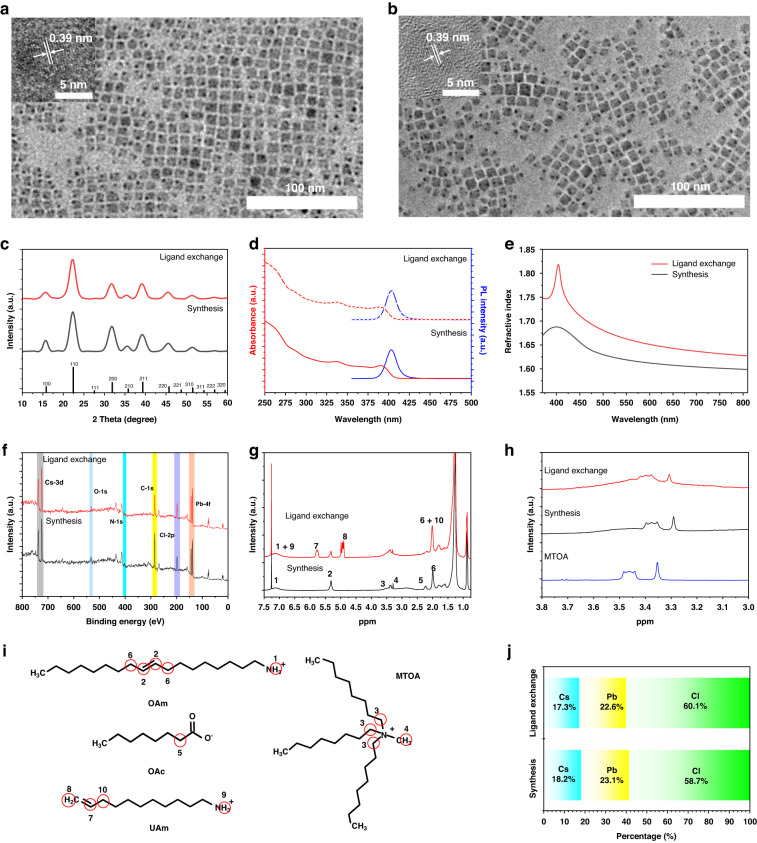
Characterizations and surface chemistry of CsPbCl_3_ PNCs. The TEM images of CsPbCl_3_ PNCs before (**a**) and after (**b**) ligand exchange process. The insert images are the high-resolution TEM of the corresponding PNCs. (**c**)The XRD patterns of the CsPbCl_3_ NCs before/after ligand exchange and the corresponding standard XRD card (PDF#73-0692). The photoluminescence emission, UV-Vis absorption spectra (**d**) and refractive index (**e**) of the CsPbCl_3_ PNCs before/after ligand exchange. **f** The XPS analysis of the CsPbCl_3_ PNCs before and after ligand exchange. **g** The ^1^H NMR characterizations of the CsPbCl_3_ PNCs before/after ligand exchange. **h** The locally enlarged ^1^H NMR characterizations of the MTOA and CsPbCl_3_ PNCs before/after ligand exchange. **i** The structures of the ligands bonding on the CsPbCl_3_ PNCs surface and the position circled in red are labeled by numbers corresponding to the resonance sources in (**g**). **j** The atomic ratios of the CsPbCl_3_ PNCs before/after ligand exchange obtained from elemental analysis by EDS

The CsPbCl_3_ PNCs synthesis was conducted at room temperature. We first utilized methyltrioctylammonium chloride (MTOA) to make PbCl_2_ dissolve in p-xylene as a Pb-precursor. The cesium octanoate (CsOAc) was injected into Pb-precursor under stirring, during which CsPbCl_3_ PNCs nucleated and grew. After a while, long-chain ligand oleylamine (OAm) was added to ensure the stability and dispersibility of CsPbCl_3_ PNCs. Then, the surface of as-synthesized pristine PNCs was modified with EAm through a ligand exchange process, after which the PNCs can well disperse in styrene (St), as shown in Supplementary Fig. S1. The pristine PNCs exhibit an average size of about 6–10 nm with distinct lattice fringe of 0.39 nm corresponding to (110) plane (PDF#73-0692) and the modified PNCs exhibit almost the same size. (Fig. 1a, b) The X-ray diffraction (XRD) pattern further confirms both pristine and modified PNCs possess a cubic crystalline perovskite structure (space group: Pm-3m). (Fig. 1c) From the optical spectra of Fig. 1d, both show excitonic absorption peaks at 390 nm and emission peaks at 403 nm with narrow line width of about 15 nm. The refractive index of modified PNCs is much higher than that of pristine PNCs (Fig. 1e), which is attributed to the reduction of ligand content as evidenced by thermogravimetric analysis (TGA) in Supplementary Fig. S2a, b^47^.

### Surface chemistry of CsPbCl_3_ PNCs

Investigating the PNC surface chemistry is critical for hybridization with PS. Therefore, ^1^H nuclear magnetic resonance (NMR), X-ray photoelectron spectroscopy (XPS) and Energy dispersive spectrometer (EDS) were conducted to identify the PNC surface. The XPS analysis (Fig. 1f) shows no significant changes in core levels after the ligand exchange process and the corresponding high-resolution XPS spectra of Cs 3d, Pb 4 f, Cl 2p and N 1 s are presented in Supplementary Fig. S3a–d. The N 1 s and O 1 s core level signals of XPS in combination with the ^1^H NMR characterization (Fig. 1g) demonstrate that all the three ligands (OAm, MTOA, OAc) exist on the surface of pristine PNCs. All the sources of the resonances are marked in Fig. 1i. The reference ^1^H NMR spectra of the ligands are shown in Supplementary Fig. S4. Ligands bonding to the surface will feature broadened and shifted resonances^4^. The broad resonance 1 ascribes to the protonated amine group (-NH_3_^+^) of protonated OAm (Fig. 1g), which can further be demonstrated by the N 1 s core level in Supplementary Fig. S3d. As N 1 s signal around 402 eV is ascribed to -NH_3_^+^ and there is no signal around 400 eV for amine group (-NH_2_)^48^. Compared with the reference ^1^H NMR spectrum of OAc, the resonance 5 assigned to OAc has slightly shifted from 2.35 ppm to 2.23 ppm caused by deprotonation. Therefore, OAm is protonated with OAc to form oleylammonium octanoate bonding to the PNCs surface, which is coincident with the conclusion of Kovalenko group^4^. As for MTOA, it is also a bound ligand as the resonances 3 and 4 are obviously broadened with slight shifts and can still be detected on the modified PNCs surface (Fig. 1h). The EAm tightly bonds on the modified PNCs surface after ligand exchange treatment demonstrated by the characteristic resonance 7 and 8. The relative molar amount of the main ligand can be calculated roughly, which is EAm: OAm: MTOA = 1: 0.58: 0.36. (Details shown in Supplementary Note 1) The atomic ratio Cs: Pb: Cl of pristine and modified PNCs is 1: 1.27: 3.23 and 1: 1.30: 3.47 (Fig. 1j). After modification, the ratios of Pb/Cs and Cl/Cs are much higher than the theoretical value, indicating that the PNCs have a Cs-poor surface. We ascribe the formation of the Cs-poor surface to the Cs-poor synthesis condition as Cs: Pb: Cl = 1: 4.5: 18.

### The fabrication of PNCs/PS nanocomposites

**Fig. 2:**
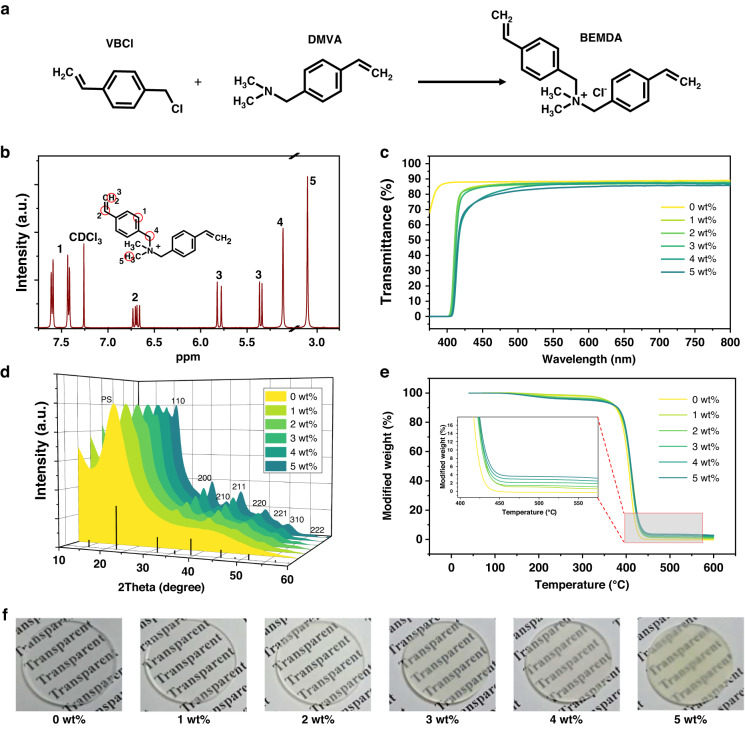
Synthesis of BEMDA and characteristics of CsPbCl_3_ PNCs/PS nanocomposites. **a** The synthesis route of the BEMDA. **b** The ^1^H NMR characterization of BEMDA. The position of the BEMDA circled in red are labeled by numbers corresponding to resonance sources. **c** The transmittance spectra of the CsPbCl_3_ PNCs/PS nanocomposites. All the samples were tested in 2 mm thickness. **d** The XRD patterns of CsPbCl_3_ PNCs/PS nanocomposites with different doping content and the standard XRD card of CsPbCl_3_. (PDF#73-0692). **e** The TGA of the CsPbCl_3_ PNCs/PS nanocomposites with different doping content and the insert is the enlarged view. **f** The photographs of the wafers with 2 mm thickness fabricated by CsPbCl_3_ PNCs/PS nanocomposites with different doping content

Though EAm can help PNCs disperse in St, the EAm has an extremely low polymerization activity, resulting in the precipitation of the modified PNCs during a subsequent polymerization process. (Supplementary Fig. S5a, b) To enhance the compatibility of PNCs with PS, we synthesized a kind of quaternary ammonium salt (BEMDA) with high polymerization activity as another kind of surfactant shown in Fig. 2a and the corresponding NMR analysis is shown in Fig. 2b. The nanocomposites can still maintain a high level of transmittance when the doping content is up to 3 wt%. (Fig. 2c) The high transmittance can be ascribed to the even distribution of PNCs in PS as shown in TEM after ultra-thin section treatment (Supplementary Fig. S6a–e) and the PNCs still show a small size after hybridization. When the particle size is much less than the light wavelength, the light scattering by the particles can be described by Rayleigh scattering theory^49–51^, which accounts for the high transmittance of the nanocomposites. Typically, the size range of the particle is less than one-tenth of the light wavelength^52,53^. When the doping content increased to 4 wt%, the PNCs begin to show a slight aggregation, therefore the transmittance decreases slightly. From the XRD pattern (Fig. 2d), the diffraction intensity gradually increases as the doping content increases from 0 wt% to 5 wt% and the crystalline structure of PNCs remains unchanged. To identify the doping content, we conducted a thermogravimetric (TGA) analysis (Fig. 2e and Supplementary Table S1), which shows negligible differences between theoretical and actual doping content. The photographs of bulk wafers fabricated by CsPbCl_3_ PNCs/PS nanocomposites with different doping content are shown in Fig. 2f.

### Surface illuminance performance of LGP based on PNCs/PS nanocomposites

**Fig. 3:**
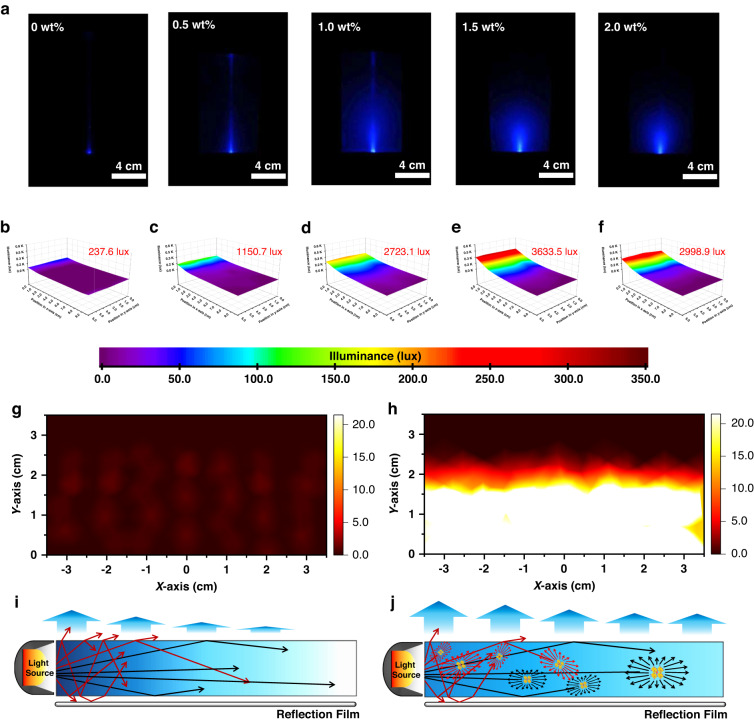
The performance of LGP with different doping content and FEA qualitative simulation. **a** The photographs of bulk CsPbCl_3_ PNCs/PS nanocomposites with a blue laser light entering from the bottom for doping content at 0 wt%, 0.5 wt%, 1.0 wt%, 1.5 wt% and 2.0 wt%. The surface illuminance of LGPs with doping contents of 0.0 wt% (**b**), 0.5 wt% (**c**), 1.0 wt% (**d**), 1.5 wt% (**e**), and 2.0 wt% (**f**). The color scale under the bottom displays the color changes with the illuminance. The surface luminance of LGP without (**g**) and with (**h**) PNCs doping obtained from FEA qualitative simulation. The schematic of the light conducted in LGP without (**i**) and with (**j**) PNCs as scatters. The light satisfying the law of total internal reflection is shown in the black line and the red line indicates the light doesn’t satisfy the law of total internal reflection

As PS is an important kind of optical waveguide material due to its high refractive index^54^, we found the CsPbCl_3_ PNCs with higher refractive index can scatter the light conducting in bulk PS without serious extinction due to the Rayleigh scattering property^55^. As shown in Fig. 3a we could intuitively observe the blue laser light spreads evenly in the bulk plate based on PNCs/PS nanocomposites and the surface luminance is higher than the control. Therefore, the light can keep conducting in the bulk PNCs/PS nanocomposite while enhancing the surface light output without severe loss, indicating a huge potential to serve as LGP in LCD application.

To show the performance of the LGPs, we fabricated large-size bulk nanocomposites up to 5.0 inches with doping contents at 0, 0.5, 1.0, 1.5, and 2.0 wt%. For convenience, the LGP with N wt% doping content is shown in N wt%-LGP. We conducted a surface illuminance test with a blue edge-light source on the short side of the LGP. The test area is at the central large visible area. (Details shown in Supplementary Fig. S7) The illuminance on the surface of the LGP is much higher than that of the control. (Fig. 3b–f) The illuminance of 1.5 wt%-LGP can even reach 3633.5 lux about 15.3 times higher than that of the 0 wt%-LGP. However, when the doping content becomes further higher, the surface illuminance begins to decrease because most of the light is consumed near the light incident edge. The enhancement of the surface illuminance is based on the consumption of the light conducted in the LGP caused by the scattering of the PNCs. When the doping content becomes further higher, the scattering ability of the LGP becomes further higher, correspondingly, most of the light is consumed and output from the light incident edge (as shown in Supplementary Fig. S7 marked as light blue) before it reaches the center display area (marked in dark blue). Therefore, the excellent performance of LGP depends not only on the total illuminance but also on the uniformity, which means the enhancement of the surface luminance should not be at the severe expense of the conduction distance. Though the 1 wt%-LGP shows a moderate surface illuminance, it shows a slow decay rate as the illuminance can still be detected after 10 cm distance conduction, which means a low illuminance difference in practical display. (Supplementary Fig. S8).

Qualitative simulation based on finite element analysis (FEA) was conducted to show the effect of PNCs on the light. The light conducting in the LGP can be divided into two parts: the light meets the condition for total internal reflection (*L*_1_) and the light does not (*L*_2_). For the light *L*_1_, the light will conduct in LGP and will not contribute to the surface illuminance. (Supplementary Fig. S9a) As for the light *L*_2_, the light will output unevenly from the surface near the light source. (Supplementary Fig. S9b) In this situation, the on-surface output light will exhibit a low luminance and poor uniformity. (Fig. 3g) However, when there are PNCs inside the LGP, the light *L*_1_ will contribute to the surface luminance (Supplementary Fig. S9c) and the surface luminance from the light *L*_2_ can be uniformized and enhanced (Supplementary Fig. S9d). Therefore, the LGP with PNCs doping shows higher brightness and uniformity than the LGP without PNCs doping. (Fig. 3h) We illustrate the PNCs scattering effect on the light in Fig. 3i, j. The light scattering is according to the Rayleigh scattering.

**Fig. 4:**
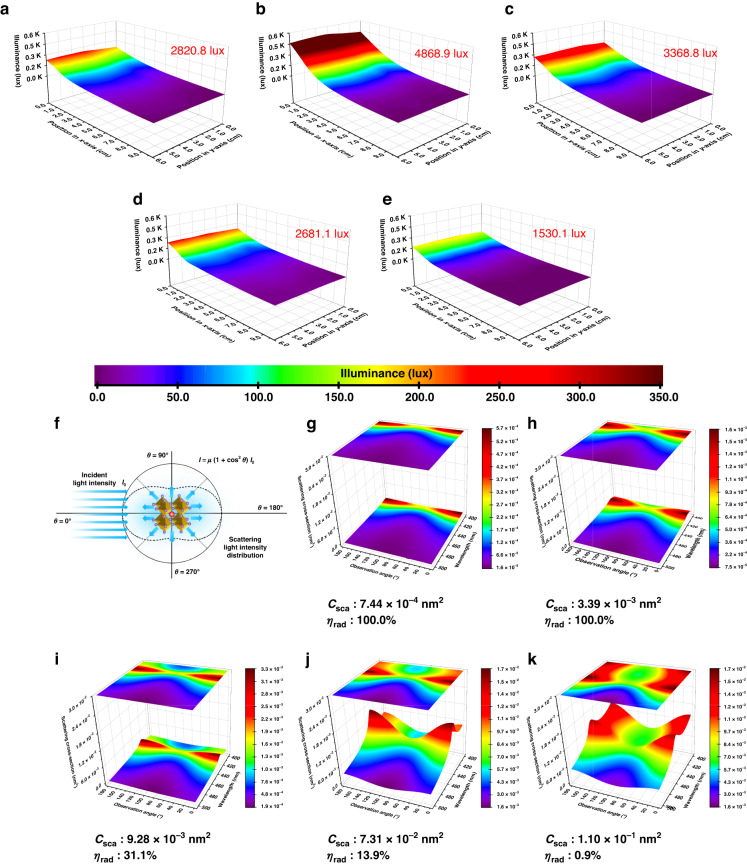
The performance of LGP with different doping component and scattering cross-section of CsPbCl_*x*_Br_3-*x*_ (1 ≤ *x* ≤ 3) PNCs. The surface illuminance of LGPs doping with CsPbCl_3_ (**a**), CsPbCl_2.5_Br_0.5_ (**b**), CsPbCl_2.0_Br_1.0_ (**c**), CsPbCl_1.5_Br_1.5_ (**d**), and CsPbCl_1.0_Br_2.0_ (**e**) PNCs. The color scale under the bottom displays the color changes with the illuminance. **f** The schematic diagram of the light distribution scattered by a single PNC. The equation shows the relationship between the incident light intensity I_0_ and the scattering light intensity I and the μ can be regarded as a constant for a certain condition. The variations of scattering cross-section per unit solid angle of CsPbCl_3_ (**g**), CsPbCl_2.5_Br_0.5_ (**h**), CsPbCl_2.0_Br_1.0_ (**i**), CsPbCl_1.5_Br_1.5_ (**j**), and CsPbCl_1.0_Br_2.0_ (**k**) PNCs with observation angle and wavelength

We extended this strategy to other PNC compositions as the optical properties can be adjusted facilely through composition adjustment. The styrene-soluble CsPbCl_*x*_Br_3-*x*_ (*x* = 1.0, 1.5, 2.0, 2.5, 3.0) PNCs show proper optical characteristics for the conducting light (Supplementary Fig. S10a) and the refractive index gradually increases as the Br component increases. (Supplementary Fig. S10b) The characteristics of the absorption coefficient obtained from spectroscopic ellipsometry are consistent with the absorption spectra demonstrating the veracities of refractive index and absorption coefficient. The refractive indexes are 1.70, 1.74, 1.83, 1.91, 2.02 at 450 nm for CsPbCl_*x*_Br_3-*x*_ (*x* = 1.0, 1.5, 2.0, 2.5, 3.0) PNCs, respectively. As Br content increases, the CsPbCl_*x*_Br_3-*x*_ PNCs still maintain a cubic crystalline perovskite structure (space group: Pm-3m) with a slight diffraction peak position shift caused by enlarged interplanar crystal spacing (Supplementary Fig. S10c) and the average crystal size shows a little bit increment from 8 to 11.5 nm. (Supplementary Fig. S11a–f) The XPS spectra of CsPbCl_*x*_Br_3-*x*_ PNCs are also shown in Supplementary Fig. S12. The nanocomposites doping with 1 wt% CsPbCl_*x*_Br_3-*x*_ PNCs still show a high transmittance (Supplementary Fig. S13a, b), and the PNCs uniformly disperse in the polymer matrix without aggregation demonstrating the universality of the hybridization method. (Supplementary Fig. S14) We also conducted the surface illuminance test to LGP doping with CsPbCl_*x*_Br_3-*x*_ PNCs (x-LGP). (Fig. 4a–e) The 2.5-LGP shows the best surface illuminance up to 4868.9 lux about 20.5 times higher than the control LGP in Fig. 3b. The total illuminance will not further improve when the Br content of the PNCs becomes further high due to an obvious absorption of CsPbCl_1.5_Br_1.5_ and CsPbCl_1.0_Br_2.0_ PNCs.

The performance of LGP is affected by several factors, mainly including the scatter and absorption of light by PNCs and PNC concentration^49,51,56^. As for the fluorescence of PNCs, multiple reabsorption processes will result in a negligible influence on the on-surface luminance^16^. For a single small PNC, light scattering can be described by Rayleigh scattering theory^50^. Unlike Mie scattering, whose scattering is directional and occurs in narrow angles^57^, the Rayleigh scattering can be regarded as “isotropic scattering” and the light scattered by PNC distributes according to $1+{\cos }^{2}\theta$, where the $\theta$ is the observation angle. (Fig. 4f) Therefore, the intensity of the light scattered in the nearly horizontal direction is higher than that in the nearly vertical direction, which means the PNCs can not only make the light homogenously distribute but also enhance the on-surface luminance. Figure 4g–k illustrates the variations of scattering cross-section per unit solid angle of CsPbCl_*x*_Br_3-*x*_ PNCs with observation angle and wavelength. All the related calculation details are shown in Supplementary Note 2 and Supplementary Table S2. The scattering cross-section per unit solid angle generally increases and the maximum value for different CsPbCl_*x*_Br_3-*x*_ PNCs gradually shifts to a longer wavelength as the Br component increases. Therefore, the total scattering cross-section (*C*_sca_) is utilized to show the comprehensive scattering effect on the light source, which is defined as the weighted average of the scattering cross-section according to the intensity distribution of the light source. The *C*_sca_ also gradually increases as the Br component increases, indicating an enhanced scattering ability of PNCs to the light. For Rayleigh scattering, the *C*_sca_ of a single PNC is too small to perceive. By taking PNC concentration into account, the volume scattering coefficient (*k*_sca_) can be regarded as the ability to scatter the light in LGP per unit volume^56^. The *k*_sca_ are 3.86 × 10^−8^_,_ 1.48 × 10^−7^_,_ 2.09 × 10^−7^_,_ 1.05 × 10^−6^ and 6.86×10^−7 ^nm^−1^ for x-LGP (*x* = 3.0, 2.5, 2.0, 1.5, 1.0), respectively. Whereas, the absorption of the PNCs cannot be ignored, especially for CsPbCl_*x*_Br_3-*x*_ (1.0 ≤ *x* ≤ 2.0) PNCs. Optical radiation efficiency (*η*_rad_) represents the fraction of scattering light out of the extinction, which shows a comparison of the scattering and the absorption effect on the light^56,58^. The *η*_rad_ of CsPbCl_3_ and CsPbCl_2.5_Br_0.5_ PNCs are both 100.0%, which means the illuminance is only affected by *k*_sca_. Therefore, 2.5-LGP shows a higher illuminance than 3-LGP. The *η*_rad_ of CsPbCl_2.0_Br_1.0_ PNCs begins to decrease, leading to a reduction of illuminance for 2-LGP. But when compared with 3-LGP, the comprehensive influence of CsPbCl_2.0_Br_1.0_ PNCs is still positive to the illuminance due to the higher *k*_sca_. The illuminance of 1.5-LGP drops to 2681.1 lux even lower than 3-LGP caused by the significant reduction of *η*_rad_. When the ratio of Br: Cl becomes further higher, the *η*_rad_ becomes extremely low up to 0.73% and most of the light is absorbed by CsPbCl_1.0_Br_2.0_ PNCs, leading to a serious loss of illuminance. The spectra of the light output from different LGPs are shown in Supplementary Fig. S15. The spectrum of the output light began to show an obvious change from LGP doping with CsPbCl_1.5_Br_1.5_ PNCs as there is a notable absence of light <430 nm. When the ratio of Br becomes further high, the optical peak even changes from 450 to 463 nm. The changes of the optical spectra are mainly attributed to the absorption of the PNCs inside the LGP.

### Performance in liquid crystal display

**Fig. 5:**
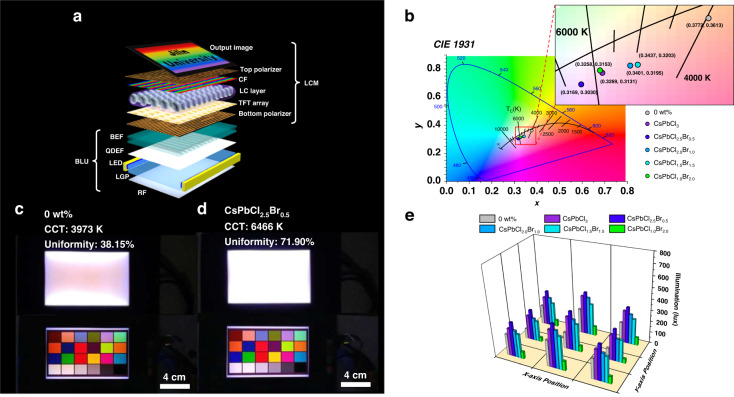
The performance of LGP in liquid crystal display. **a** The device configuration of the LCD display. The LCD module is about 5.0 inches in size. The LCD module consists of two parts: liquid crystal module (LCM) and backlight unit (BLU). The LCM mainly includes polarizers, liquid crystal (LC) materials with a thin-film transistor (TFT) array and color filters (CF). The LGP equipped with light emitting diode (LED) strips on the sides and a series of optical films constitute the BLU. The optical films include reflection film (RF), quantum dot enhanced film (QDEF) and brightness enhancement film (BEF). **b** The CIE 1931 diagram at the central region of the LCD screen based on the control LGP with no PNCs doping (0 wt%) and LGPs doping with CsPbCl_*x*_Br_3-*x*_ (1≤ *x* ≤ 3) PNCs under white light operating condition. The corresponding chromaticity coordinates are marked in the partially enlarged view. The photographs of the BLU (top) and the LCD (bottom) under operation based on the control LGP without PNCs (**c**) and the LGP doping with CsPbCl_2.5_Br_0.5_ PNCs (**d**). **e** The results of the LGP uniformity test by the nine-point method according to the SJ/T 11348-2006 standard

We explored the commercial potential by integrating the LGP with advanced display technology^59,60^. The device configuration is typical (Fig. 5a), where the blue edge-light source is converted into surface light by LGP and passes through the quantum dot enhanced film (QDEF) and brightness enhancement film (BEF) for color conversion and brightness enhancement. Finally, the liquid crystal module (LCM) controls the illuminating light to show various images. The CIE 1931 diagram (Fig. 5b) shows all the chromaticity coordinates of the LCDs under white light operating mode. The correlated color temperature (CCT) of the LCD generally increases with the surface illuminance of LGP as the blue light component increases (Supplementary Fig. S16). While the CCT based on 1.0-LGP is abnormally higher than that based on 3.0-LGP, we ascribe it to the obviously changed optical distribution of output light (Supplementary Fig. S15). The peak of the output light is changed from 450 nm to 465 nm and there is almost no light distribution <440 nm due to the absorption of the CsPbCl_1.0_Br_2.0_ PNCs. The changed optical distribution will influence the light conversion of QDEF. Therefore, we further characterized the absorption and fluorescence spectrum of the QDEF (Supplementary Fig. S17). The result of the uniformity test according to SJ/T 11348-2006 standard is shown in Fig. 5e and details are presented in Supplementary Fig. S18 and Supplementary Table S3. The uniformities under lab level are 38.15%, 63.32%, 71.90%, 59.10%, 62.78%, and 45.55% for the control, 3.0, 2.5, 2.0, 1.5, and 1.0-LGP, respectively. All the uniformities based on LGP show obvious improvement. Figure 5c, d show the backlight unit (BLU) and LCD quality based on the control and the best-performing 2.5-LGP. For other LGPs, the photographs of display quality are shown in Supplementary Fig. S19. The control BLU exhibits an obvious difference between the central region and the edge region. However, the performance of BLU based on 2.5-LGP shows a huge promotion both in illuminance and uniformity. The improvement in luminance also means a reduction in electric power consumption as LGP equipped with a low-intensity light source can achieve a better surface luminance than a normal LGP equipped with a very high-consumption light source does. To test the stability of the LGP, we first measured the fluorescence spectra of the nanocomposites with 1 wt% as shown in Supplementary Fig. S20a. Based on the characterization, we further observed the photoluminescence (PL) intensity changes of nanocomposite doping with CsPbCl_1.0_Br_2.0_ PNCs under 50 W UV-irradiation and in water to show the UV and humidity stability, respectively. As shown in Supplementary Fig. S20b, c, the PL intensity only dropped by roughly 20% under continuous irradiation with UV light for about 100 h and there is almost no change of the PL intensity for the sample immersing in water, indicating a high humidity resistance. The excellent performance of LGP in display quality and energy consumption shows a huge potential in practical application.

## Discussion

In summary, we successfully fabricated bulk CsPbCl_x_Br_3-x_ PNCs/PS nanocomposites by a two-type ligand strategy, where the PNCs disperse uniformly in the polymer matrix without aggregation. Further, the Rayleigh scattering behavior of the nanocomposites can be adjusted by the PNC composition adjustment. Based on this property, we first used PNCs as a kind of scatters inside LGP, which could improve the illuminance and uniformity at the same time. We believe this kind of LGP has huge potential in LCD applications and will draw much attention in LGP-related fields, especially as a base material to combine with the advanced LGP processing technologies such as the micro-optical pattern on the bottom or the adoption of the wedge-shaped plates.

## Materials and methods

### Chemicals

Lead chloride (PbCl_2_, 99.999%), lead bromide (PbBr_2_, 99.999%) and cesium carbonate (Cs_2_CO_3_, 99.995%) were purchased from Sigma-Aldrich. Oleylamine (OAm, 90%), undec-10-en-1-amine (EAm, 95%), octanoic acid (OAc, 99%), anhydrous p-xylene (99%), methyltrioctylammonium chloride (MTOA, 97%), methyltrioctylammonium bromide (MTOABr, 98%), 4-vinylbenzyl chloride (VBCl, 98%), methyl acetate (MeOAc, 99%), 2,2’-Azobis(2,4-dimethyl)valeronitrile (ABVN, 98%) and styrene (St, 99%) were purchased from Macklin reagent. *N*,*N*-dimethyl-4-vinylaniline (DMVA,90%) was purchased from Acros Organics.

### Synthesis of CsPbCl_*x*_Br_3-*x*_ PNCs

The preparation and purification of CsPbCl_3_ PNCs were conducted at room temperature in open air. First, Cs_2_CO_3_ (1 mmol) was dissolved in OAc (10 mL) to obtain cesium octanoate (CsOAc) solution. Second, 0.9 mmol of PbCl_2_ and 1.8 mmol of MTOA were dissolved into 9 mL anhydrous p-xylene to obtain Pb-precursor solution. Then, CsOAc solution (1 mL) was swiftly injected into the as-prepared Pb-precursor under vigorous stirring. After about 30 s, OAm (1 mL) was added and stirred for 2 min. Subsequently, MeOAc was added into the crude solution with a volume ratio of 3:1. The precipitation was collected by centrifugation at 9000 rmp and re-dispersed in 3 mL hexane for ligand exchange treatment. For the synthesis of CsPbCl_*x*_Br_3-*x*_, all the processes were the same under the corresponding ratios of PbCl_2_ to PbBr_2_ and MTOA to MTOABr.

### Modification of PNCs

For ligand exchange treatment, EAm (50 μL) was added to the above PNCs dissolved with 3 mL hexane solution. After stirring for 5 min, MeOAc (12 mL) was added to precipitate the modified PNCs and centrifugated at 9000 rmp. The obtained PNCs powder was completely dry under vacuum for further use.

### Synthesis of BEMDA

The synthesis of quaternary ammonium salt is based on the previous study with some changes^61^. Briefly, 3.584 g of DMVA and 3.114 g of VBCl were mixed in acetone (60 mL). The mixture was stirred and refluxed at 45 °C overnight. The precipitated BEMDA in the mixture was purified and recrystallized by acetone/ether. The product was further washed to white by ether. ^1^H-NMR (CDCl_3_, 400 MHz): 7.26–7.61 (m, 8H, ArH), 6.66–6.73 (m, 2H, ArCH=), 5.34–5.82 (m, 4H, ArCH=CH_2_), 5.11 (s, 4H, ArCH_2_-N^+^), 3.11 (s, 6H, N^+^-CH_3_).

### Fabrication of PNCs/PS nanocomposites

The stabilizer was removed from St monomer before use. The modified PNCs with corresponding weight, BEMDA, 1 wt% ABVN were mixed in St solution. The weight of BEMDA was about one-tenth of the modified PNCs’ weight. The mixture was stirred until clear and pre-polymerized for 16 h under 50 °C. Subsequently, the viscous oligomer was injected into the mold and degassed until there are no bubbles in the system. After the injection, the mold was further heated at 60 °C for 36 h to make the polymerization complete.

### Characterizations

UV-visible absorption spectra were obtained using a Shimadzu 3600 UV-visible-NIR spectrophotometer and photoluminescence (PL) spectra on a Cary Eclipse spectrofluorimeter. Nuclear magnetic resonance spectra (NMR) were collected from a Zhongke-Niujin Nuclear magnetic resonance spectrometer 400 MHz in deuterated chloroform. The refractive index and absorption efficient were measured via a variable angle spectroscopic ellipsometer (J.A.Woollam Co., Inc., USA) with the UV-vis-NIR range from 1.24 to 4.13 eV. The ellipsometric angle ψ and phase difference Δ were recorded at incidence angles of 55, 65, and 75°, respectively. The detail morphology of the samples was observed on a JEM-2100F at an accelerating voltage of 200 kV via a transmission electron microscopy (TEM). Energy Disperse Spectroscopy (EDS) for elemental analysis was conducted on a Bruker Energy Dispersive Spectrometer based on a SU8020 electron microscope. X-ray diffraction (XRD) data were collected using a PANalytical B.V.-Empyream Diffractometer with Cu Kα radiation. X-ray photoelectron spectroscopy (XPS) were measured in an integrated ultrahigh vacuum system equipped with multitechnique surface analysis system (VG ESCALAB MK II spectrometer). The transmittance test of material was conducted on an UV-visible-NIR spectrophotometer from PerkinElmer company. Thermogravimetric analysis (TGA) was conducted on NETZSCH STA449F3 QMS403D/Bruker V70 in N_2_ atmosphere with 10 °C min^−1^ heating rate. The ultrathin section treatment to the nanocomposites was conducted by a Leica EM UC7 cryo-cut microtome. The illuminance test was conducted by an illuminometer from Suzhou TASI Electronics. The chromaticity coordinate, correlated color temperature and corresponding optical spectrum were tested by a photometer SPIC-300BW from EVERFINE.

### Qualitative simulation based on finite element analysis (FEA)

The FEA simulation was conducted based on practical condition, such as the material of LGP and the refractive index of PNCs at 450 nm. The boundary conditions of LGP were set as specular reflection on the bottom and elimination on sides. The light scattering distribution was manually controlled as Rayleigh distribution. The surface brightness was obtained by frozen wall with accumulation condition.

### Supplementary information

Supplementary Information

## Data Availability

The data that support the plots within this paper and the other findings of this study are available from the corresponding authors upon reasonable request.
