# Supplementary material for: Bulk CsPbClxBr3-x (1 ≤ x ≤ 3) perovskite nanocrystals/polystyrene nanocomposites with controlled Rayleigh scattering for light guide plate
Source: Light Sci Appl. 2023 Nov 1;12:261. doi: 10.1038/s41377-023-01306-z (PMC10620209; doi:10.1038/s41377-023-01306-z)
Supplement: Supplementary file 1 — Supplementary Information [file 41377_2023_1306_MOESM1_ESM.docx]

**Supporting information for**

**Bulk CsPbCl_x_Br_3-x_ (1≤x≤3) perovskite nanocrystals/polystyrene nanocomposites with controlled Rayleigh scattering for light guide plate**

Chongming Liu^1^, Zhicheng Zhu^1^, Kaibo Pan^1^, Yuan Fu^1^, Kai Zhang^1^ and Bai Yang^1^*

^1^State Key Laboratory of Supramolecular Structure and Materials, College of

Chemistry, Jilin University, Changchun, 130012, China.

*Corresponding author: Prof. Bai Yang ([byangchem@jlu.edu.cn](mailto:byangchem@jlu.edu.cn))

**Supplementary Note 1**

The relative amount calculation of ligands can be expressed as following equation:^1^

$$M_{EAm}:M_{OAm}:M_{MTOA}=\frac{A_{EAm}}{P_{EAm}}:\frac{A_{OAm}}{P_{OAm}}:\frac{A_{MTOA}}{P_{MTOA}}$$

(1)

where M, A and P are molar number, integral area of the quantitative chemical shift peak and the corresponding proton number of the integrated peak, respectively. The subscripts represent different ligands. The calculation is based on five times repeated characterizations. The results are listed as below:

|  | $\frac{A_{EAm}}{P_{EAm}}$ | $\frac{A_{OAm}}{P_{OAm}}$ | $\frac{A_{MTOA}}{P_{MTOA}}$ |
| --- | --- | --- | --- |
| Characterization 1 | 1.000 | 0.600 | 0.381 |
| Characterization 2 | 1.000 | 0.590 | 0.351 |
| Characterization 3 | 1.000 | 0.575 | 0.366 |
| Characterization 4 | 1.000 | 0.565 | 0.376 |
| Characterization 5 | 1.000 | 0.585 | 0.346 |
| Average | 1.000 | 0.583±0.014 | 0.364±0.015 |

Note: The chemical shift peaks of 7, 2 and 3+4 are selected to calculate the integral area of EAm, OAm and MTOA, respectively.

**Supplementary Note 2**

The scattering cross-section per unit solid angle $\sigma_{sca}(\theta)$of PNC at different observation angles ($\theta$) can be described as following:^2, 3^

$\sigma_{sca}(\theta)=\frac{1}{2}{r^{2}x^{4}\left| \frac{m^{2}-1}{m^{2}+2} \right|}^{2}\left( 1+{cos}^{2} \theta\right)$;

(2)

$$m=\frac{n}{n_{0}};$$

(3)

$x=kr$;

(4)

$$k=\frac{2\pi n_{0}}{\lambda},$$

(5)

where k is the wave number, *λ* is the wavelength of the light, *х* is the size parameter of the PNC, r refers to the size of the PNC and *m* is the relative refractive index of PNC (*n*) and medium (*n_0_*). The *θ* is a polar angle and the *dω* means the solid angle between *θ* and *θ+dθ*. For clarity, we illustrate the *θ* and *dω* in the below schematic diagram.

Their relationship can be described as

$d\omega=2\pi\sin\thetaⅆ\theta$,

(6)

Therefore, the scattering cross-section ($\sigma_{sca}$) of PNC can be calculated by

$$\sigma_{sca}=\int_{0}^{4\pi} \sigma_{sca}(\theta)ⅆ\omega=\frac{8}{3}{\pi{r^{2}x}^{4}\left| \frac{m^{2}-1}{m^{2}+2} \right|}^{2},$$

(7)

However, the$\sigma_{sca}$ varies with the wavelength. As a result, we utilize the total scattering cross-section $\sigma_{sca}$ to show the comprehensive scattering effect to the light source, which covers a range of wavelength with inhomogeneous intensity distribution. The $C_{sca}$ can be written as

$$C_{sca}=\sum_{\lambda=420}^{490} \sigma_{sca-\lambda}\cdot f_{\lambda},$$

(8)

where $f_{\lambda}$ is the intensity proportion at the corresponding wavelength λ of total light source intensity and$\sigma_{sca-\lambda}$ refers to the $\sigma_{sca}$ at λ. For Rayleigh scattering, the $C_{sca}$ of a single PNC is too small to perceive. As the PNCs distribute in the LGP randomly, separate scattered waves are incoherent and their intensity can be additive at any direction.^3^ Therefore, the volume scattering coefficient can be expressed as

$$k_{sca}=NC_{sca},$$

(9)

where N represents the PNCs per unit volume. Similarly, the volume absorption coefficient $k_{abs}$and total absorption cross-section $C_{abs}$ can be described as following:^2, 3^

$$k_{abs}=NC_{abs};$$

(10)

$$C_{abs}=\sum_{\lambda=420}^{490} \sigma_{abs-\lambda}\cdot f_{\lambda};$$

(11)

$$\sigma_{abs}=4\pi r^{2}x\mathrm{Im}\left\{ \frac{m^{2}-1}{m^{2}+2} \right\},$$

(12)

where$\sigma_{abs}$ is the absorption cross-section and$\sigma_{abs-\lambda}$ refers to the $\sigma_{abs}$ at λ. To show the relative strength of these processes, the optical radiation efficiency or albedo ($\eta_{rad}$) can be calculated by^2, 4^

$$\eta_{rad}=\frac{k_{sca}}{k_{sca}+k_{abs}}=\frac{C_{sca}}{C_{sca}+C_{abs}},$$

(13)

**Figure S1.** The schematic diagram of the synthetic process of the styrene-soluble CsPbCl_3_ PNCs. The photographs show 20 mg ml^-1^ CsPbCl_3_ PNCs in styrene solution before and after the ligand exchange process. The red laser demonstrates the dispersity of PNCs in the styrene.

**Figure S2.** The TGA of the CsPbCl_3_ PNCs before (a) and after (b) ligand exchange process.

**Figure S3.** The high-resolution XPS spectra of Cs 3d (a), Pb 4f (b), Cl 2p (c) and N 1s (d) of the CsPbCl_3_ PNCs before and after ligand exchange.

**Figure S4.** The ^1^H NMR characterizations of undec-10-1-amine, oleylamine, octanoic acid and methyltrioctylammonium chloride.

**Figure S5.** The photographs are the CsPbCl_3_ PNCs/PS nanocomposites fabricated without (a) and with (b) BEMDA.

**Figure S6.** The TEM images of CsPbCl_3_ PNCs in PS matrix at 1 wt% (a), 2 wt% (b), 3 wt% (c), 4 wt% (d), 5 wt% (e) doping content after 40 nm ultra-thin section treatment.

**Table S1.** The calculation of the actual doping content.

| Theoretical content | Residue at 525℃ | Actual content |
| --- | --- | --- |
| 1 wt% | 0.90 wt% | 1.24 wt% |
| 2 wt% | 1.33 wt% | 1.83 wt% |
| 3 wt% | 2.10 wt% | 2.89 wt% |
| 4 wt% | 2.81 wt% | 3.87 wt% |
| 5 wt% | 3.52 wt% | 4.84 wt% |

Note: The actual doping content is calculated in consideration of the PNC ligand content 27.3 wt% from Figure S2b.

**Figure S7.** The schematic (top view) of the surface illuminance test. The dark blue area is the test area. The bottom of the LGP is covered with reflection film and the sides are equipped with black mask except edge-light side.

**Figure S8.** The illuminance of LGPs with different PNC doping content along with distance variation.

**Figure S9.** The results of FEA qualitative surface luminance simulation. The results of the LGP without PNCs for the light satisfies the total reflection (a) and the light doesn’t satisfy the total reflection (b). The results of the LGP with PNCs for the light satisfies the total reflection (c) and the light doesn’t satisfy the total reflection (d).

**Figure S10.** (a) The photoluminescence and UV-Vis absorption spectra of the CsPbCl_x_Br_3-x_ (1≤x≤3) PNCs. The optical spectrum of the light source is also presented to compare the optical properties of CsPbCl_x_Br_3-x_ PNCs conveniently. (b) The refractive indexes and absorption coefficients of the CsPbCl_x_Br_3-x_ PNCs. (c) The XRD patterns of the CsPbCl_x_Br_3-x_ PNCs and the standard XRD card (PDF#73-0692).

**Figure S11.** The TEM images of the CsPbCl_3_ (a), CsPbCl_2.5_Br_0.5_ (b), CsPbCl_2.0_Br_1.0_ (c), CsPbCl_1.5_Br_1.5_ (d) and CsPbCl_1.0_Br_2.0_ (e) PNCs. (f) The size distribution histograms of CsPbCl_x_Br_3-x_ (1≤x≤3) PNCs.

**Figure S12.** The XPS spectra of Br-contained PNCs.

**Figure S13.** (a) The transmittance spectra of the CsPbCl_x_Br_3-x_ (1≤x≤3) PNCs/PS nanocomposites. (b) The photographs of the wafers fabricated by CsPbCl_x_Br_3-x_ PNCs/PS nanocomposites.

**Figure S14.** The TEM images of the CsPbCl_3_ (a), CsPbCl_2.5_Br_0.5_ (b), CsPbCl_2.0_Br_1.0_ (c), CsPbCl_1.5_Br_1.5_ (d) and CsPbCl_1.0_Br_2.0_ (e) PNCs in PS matrix after 40 nm ultra-thin section treatment.

**Table S2.** The main parameters of the calculation in Supplementary Note 2.

| PNCs | $\boldsymbol{C}_{\boldsymbol{sca}}$(nm^2^) | $\boldsymbol{C}_{\boldsymbol{abs}}$(nm^2^) | $\boldsymbol{k}_{\boldsymbol{sca}}$(nm^-1^) | $\boldsymbol{k}_{\boldsymbol{abs}}$(nm^-1^) | $\boldsymbol{\eta}_{\boldsymbol{rad}}$ |
| --- | --- | --- | --- | --- | --- |
| CsPbCl_3_ | 7.44×10^-4^ | 0 | 3.86×10^-8^ | 0 | 100.0% |
| CsPbCl_2.5_Br_0.5_ | 3.39×10^-3^ | 0 | 1.48×10^-7^ | 0 | 100.0% |
| CsPbCl_2.0_Br_1.0_ | 9.28×10^-3^ | 2.05×10^-2^ | 2.09×10^-7^ | 4.62×10^-7^ | 31.1% |
| CsPbCl_1.5_Br_1.5_ | 7.31×10^-2^ | 4.53×10^-1^ | 1.05×10^-6^ | 6.49×10^-6^ | 13.9% |
| CsPbCl_1.0_Br_2.0_ | 1.10×10^-1^ | 1.23×10^1^ | 6.86×10^-7^ | 7.68×10^-5^ | 0.9% |

**Figure S15.** The optical spectra of on-surface output light from different LGPs doping with CsPbCl_x_Br_3-x_ (1≤x≤3) PNCs.

**Figure S16.** The spectral distributions at the central region of the LCD screens under white light operating conditions based on the control LGP with no PNCs doping (a) and LGPs doping with CsPbCl_3_ (b), CsPbCl_2.5_Br_0.5_ (c), CsPbCl_2.0_Br_1.0_ (d), CsPbCl_1.5_Br_1.5_ (e) and CsPbCl_1.0_Br_2.0_ (f) PNCs.

**Figure S17.** The absorption (a) and fluorescence (b) spectrum of the QDEF. The absorption intensity of the QDEF is almost no difference between the 400 nm and 500 nm. However, the fluorescence of the QDEF depends on the excitation wavelength. The spectrum distribution of the light output from the 1.0-LGP is apparently different from others. Therefore, the intensity of the converted light becomes lower, leading to a higher proportion of the blue light and an abnormally higher CCT.

**Figure S18.** The schematic diagram (top view) of the uniformity test by the nine-point method according to the SJ/T 11348-2006 standard. The dark blue points refer to the selected areas to test the illuminance of the LGP.

**Table S3.** The results of the uniformity test.

| Sample | Minimum illuminance (lux) | Maximum illuminance (lux) | Uniformity |
| --- | --- | --- | --- |
| The control | 90.90±2.15 | 238.26±1.42 | 38.15% |
| CsPbCl_3_ | 224.26±1.02 | 354.12±1.90 | 63.32% |
| CsPbCl_2.5_Br_0.5_ | 282.50±1.47 | 392.86±2.16 | 71.90% |
| CsPbCl_2.0_Br_1.0_ | 204.82±1.17 | 346.56±2.45 | 59.10% |
| CsPbCl_1.5_Br_1.5_ | 187.24±1.85 | 298.26±2.08 | 62.78% |
| CsPbCl_1.0_Br_2.0_ | 39.16±1.10 | 85.98±1.78 | 45.55% |

Note: The illuminance uniformity of the LGP can be calculated by the equation 14:

$$Uniformity=\frac{Lmin}{Lmax}\times100\%$$

(14)

where $Lmin$ and $Lmax$ are the minimum and maximum illuminance values of the nine dark blue points in Figure S18, respectively.

**Figure S19.** The photographs of the BLU (top) and the LCD (bottom) under operation based on the LGP doping with CsPbCl_3_ (a), CsPbCl_2.0_Br_1.0_ (b), CsPbCl_1.5_Br_1.5_ (c) and CsPbCl_1.0_Br_2.0_ (d) PNCs.

**Figure S20.** (a) The fluorescence spectra of the nanocomposites with 1 wt% doping content under 400 nm excitation. The shoulder peak located at around 465 nm don’t change along with the change of PNCs component, indicating it is from the polymer. The BEMDA can serve as cross-linking agent and polymer chains are increasingly interconnected by phenyl groups, leading to a change in the structure of the polystyrene and corresponding intrinsic fluorescence from the phenyl groups.^5, 6^ The stability test of the CsPbCl_1.0_Br_2.0_ PNCs/PS nanocomposite under UV irradiation (b) and immersing in water (c).

**Supplementary references**

1.	Liu, C. et al. Surface ligands management for efficient CsPbBrI_2_ perovskite nanocrystal solar cells. *Sol. RRL* **4**, 2000102 (2020).

2.	Bohren, C. F. et al. *Absorption And Scattering Of Light By Small Particles.* (John Wiley & Sons, New York, USA. 2008).

3.	McCartney, E. J. *Optics Of The Atmosphere: Scattering By Molecules And Particles.* (John Wiley & Sons, New York, USA. 1976).

4.	Tanabe, K. Optical radiation efficiencies of metal nanoparticles for optoelectronic applications. *Mater. Lett.* **61**, 4573-4575 (2007).

5.	Mundra, M. et al. Fluorescence studies of confinement in polymer films and nanocomposites: Glass transition temperature, plasticizer effects, and sensitivity to stress relaxation and local polarity. *Eur. Phys. J.: Spec. Top.* **141**, 143-151 (2007).

6.	Basile, L. J. Effect of styrene monomer on the fluorescence properties of polystyrene. *J. Chem. Phys.* **36**, 2204-2210 (1962).
